# Structural Characterization, Antioxidant and Antitumor Activities of the Two Novel Exopolysaccharides Produced by *Debaryomyces hansenii* DH-1

**DOI:** 10.3390/ijms24010335

**Published:** 2022-12-25

**Authors:** Yajing Yang, Guoqiang Chen, Xiaoqi Zhao, Xiaohe Cao, Lei Wang, Jingjiu Mu, Fenghui Qi, Lijuan Liu, Haibo Zhang

**Affiliations:** 1 College of Life Sciences, Northeast Forestry University, Harbin 150040, China; 2 CAS Key Laboratory of Biobased Materials, Qingdao Institute of Bioenergy and Bioprocess Technology, Chinese Academy of Sciences, Qingdao 266101, China; 3 Shandong Energy Institute, Qingdao 266101, China; 4 Qingdao New Energy Shandong Laboratory, Qingdao 266101, China

**Keywords:** *Debaryomyces hansenii*, exopolysaccharides, structure analysis, antioxidant activity, antitumor activity, thermal property

## Abstract

Exopolysaccharides produced by edible microorganisms exhibit excellent constructive physicochemical and significant biological activity, which provide advantages for the food or pharmaceutical industries. Two novel exopolysaccharides produced by *Debaryomyces hansenii* DH-1 were characterized, named S1 and S2, respectively. S1, with a molecular weight of 34.594 kDa, primarily consisted of mannose and glucose in a molar ratio of 12.19:1.00, which contained a backbone fragment of α-D-Manp-(1→4)-α-D-Manp-(1→2)-α-D-Glcp-(1→3)-α-D-Manp-(1→3)-β-D-Glcp-(1→4)-β-D-Manp-(1→. S2, with a molecular weight of 24.657 kDa, was mainly composed of mannose and galactose in a molar ratio of 4.00:1.00, which had a backbone fragment of α-D-Manp-(1→6)-β-D-Manp-(1→2)-α-D-Manp-(1→4)-α-D-Galp-(1→3)-β-D-Manp-(1→6)-α-D-Manp-(1→. Both S1 and S2 exhibited good thermal stability and potent hydroxyl radical scavenging activity, with ~98%. Moreover, S1 possessed an additional strong iron-reducing capacity. In vitro antitumor assays showed that S1 and S2 significantly inhibited the proliferation of Hela, HepG2, and PC-9 cancer cells. Moreover, PC-9 was more sensitive to S1 compared with S2. The above results indicate that S1 and S2 have great potential to be utilized as natural antioxidants and candidates for cancer treatment in the food and pharmaceutical industries.

## 1. Introduction

Reactive oxygen species (ROS) are critical for sustaining the immune system and the redox balance [1,2]. Excessive ROS accumulation can induce oxidative damage to macromolecules and cause various diseases, such as diabetes, cardiovascular diseases, and atherosclerosis [3,4]. Cancer, as a major public health problem, is extremely devastating and has led to high mortality rates [4,5,6]. To date, synthetic antioxidants and anticancer drugs have been associated with some toxicity, resistance, or other side effects [7]. Therefore, it is necessary to develop antioxidant and anticancer drugs with high efficacy and low toxicity from natural sources. Microbial exopolysaccharides (EPSs), as natural secondary metabolites, exhibit significant biological activity [1,8,9,10], which has attracted great scientific interest, since they can be industrialized by submerged fermentation and have easy extraction procedures [11,12]. Yeast EPSs, as a significant component of microbial EPSs, have shown promising antitumor activity and possess great selectivity, as well as outstanding antioxidant activity, with a wide range of applications in the food or pharmaceutical industries [13,14,15]. The exopolysaccharide REPS-A produced by *Rhodotorula mucilaginosa* CICC 33013 exhibits appreciable free radical scavenging activity and antitumor activity against the HepG2 cell line [14], and the exopolysaccharide obtained from *Rhodotorula mucilaginosa* sp. GUMS16 displays strong 1-diphenyl-2-picrylhydrazyl (DPPH) scavenging ability [15]. The exopolysaccharides produced by *Cryptococcus heimaeyensis* S20 significantly inhibit the proliferation of NSCLC cells [16]. However, there is little research about the EPS with antioxidant and antitumor activity of *Debaryomyces hansenii* (*D. Hansenii*), which has been widely used in both basic and applied research as a significant non-infectious yeast [17,18]. It has been reported to be effective against antibiotic-related diarrhea [19] and capable of inhibiting the growth of pathogenic *Candida* yeasts [20]. Thus, the exploration of the structural characterization and functional activity of EPSs from *D. Hansenii* further enriches the information about yeast EPSs.

It is worth noting that EPSs produced by pathogenic microorganisms are at risk of contamination with endotoxins [11,21]. Once employed in the food and pharmaceutical industries, they can have display toxic side effects. Therefore, it is necessary to screen for edible microorganisms capable of producing food-grade EPSs. Using fermented foods as a source of microbial screening is a particularly sensible approach due to their abundant microbial resources and safety [11,22].

In this study, we screened and isolated a strain of *D. Hansenii* DH-1 from sausage, and extracted two novel exopolysaccharides. Antioxidant and antitumor activities were evaluated in vitro, and their structures were characterized. This study will provide valuable information on the utilization of yeast EPSs and a theoretical basis for practical applications in the food or medical fields.

## 2. Results and Discussion

### 2.1. Isolation and Identification of the Strain

**Figure 1:**
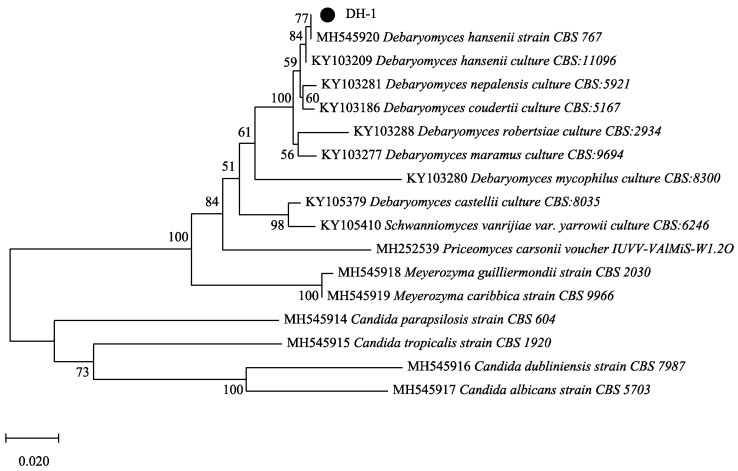
The phylogenetic tree of the strain *D. Hansenii* DH-1. Numbers at branches represent bootstrap values (above 50%, 1000 replicates) of the neighbor-joining analysis.

The strain was obtained by the plate dilution method and named *D. Hansenii* DH-1(named DH-1) by rDNA-ITS sequence analysis. On the agar plate, the colony surface of the DH-1 strain was smooth, and the edge was complete, round, or oval, with white mucus (Figure S1). The 5.8S rDNA analysis showed that the DH-1 strain had 100% identity with some *Debaryomyces hansenii* strains. The phylogenetic tree indicated that the DH-1 strain and *D. Hansenii* strain CBS 767 had the closest genetic relationship (Figure 1).

### 2.2. Purification and Composition Analysis of EPS

**Figure 2:**
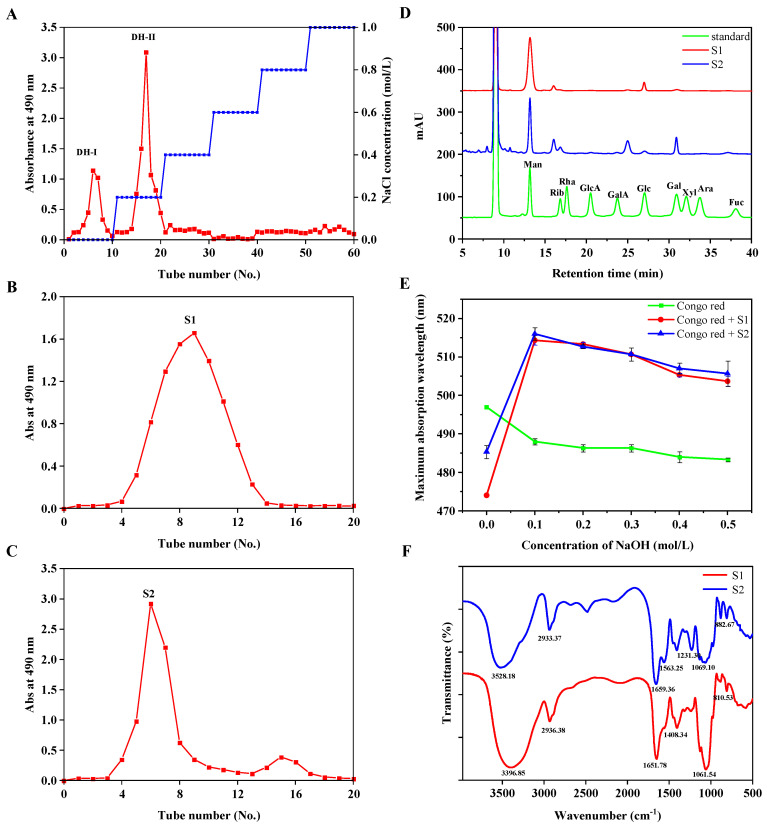
Column chromatographic separation and composition analysis. The crude EPS was subjected to gradient elution by DEAE sepharose cellulose fast-flow column (**A**), the red line represented the absorbance at 490 nm, and the blue line represented the concentration of NaCl. Two main peaks (DH−I, DH−II) were further purified by a Sephacryal S-100 column, obtaining two components named S1 (**B**) and S2 (**C**), respectively. (**D**) The HPLC chromatograms of the monosaccharide composition and peak identity. Man: Mannose; Rib: Ribose; Rha: Rhamnose; GlcA: Glucuronic acid; GalA: Galacturonic acid; Glc: Glucose; Gal: Galactose. Xyl: Xylose; Ara: Arabinose; Fuc: Fucose. (**E**) The maximum absorption wavelength of Congo red and polysaccharide samples at various concentrations of NaOH. (**F**) FT-IR spectrum of EPSs.

The extraction of crude EPS from the *D. hansenii* DH-1 fermentation broth was 1.47 ± 0.09 g/L. The crude EPS was eluted by deionized water and 0.2 M NaCl using DEAE sepharose fast-flow column chromatography, respectively, and the two fractions (named DH−I and DH−II) were obtained (Figure 2A). After further purification using a Sephacryal S-100 column, the major exopolysaccharides of S1 and S2 were collected in subsequent research (Figure 2B,C). Finally, the exopolysaccharides S1 and S2 were obtained at 0.46 ± 0.12 g/L and 0.81± 0.18 g/L, respectively. They exhibited lower yields compared to the most commonly reported exopolysaccharide. The purity of S1 and S2 reached 95.18% and 92.05% according to the method of phenol–sulfuric acid colorimetry, respectively. HPGPC chromatography indicated that the Mws of S1 and S2 were 34.594 kDa and 24.657 kDa, respectively (Table S1).

The monosaccharide composition of EPS was analyzed by PMP pre-column derivatization and HPLC. The analysis suggested that S1 primarily consisted of mannose (89.07%) and glucose (7.31%), with a molar ratio of 12.19:1.00, respectively, and S2 was mainly composed of mannose (74.42%) and galactose (18.61%), with a molar ratio of 4.00:1.00, respectively (Figure 2D). In addition, a small amount of glucuronic acid (1.00%) and galacturonic acid (1.52%) was also detected in S2. However, neither glucuronic acid nor galacturonic acid was detected in S1, indicating that S1 is a neutral polysaccharide. They were different from those of the reported polysaccharides isolated from *D. Hansenii* [23,24], and it was suggested that S1 and S2 are novel polysaccharides.

Figure 2E shows the *λ*_max_ of the complex at different concentrations of NaOH solution. The *λ*_max_ of the complex had obvious red shifts, ascribed to the formation of complexes in 0.1 M NaOH solutions. As the concentration of NaOH increased, the *λ*_max_ of the complex decreased, which indicated that the triple helix conformation translated into a single coil conformation [25]. It indicated that the two novel exopolysaccharides possessed triple helix structures, which could affect the biological activity and function of polysaccharides.

Functional groups of EPS were analyzed (Figure 2F). The intense absorption peaks at 3396.85 cm^−1^ and 3528.18 cm^−1^ corresponded to the stretching vibrations of –OH [26]. The peaks at 2933.37 cm^−1^ and 2936.38 cm^−1^ were related to the C–H stretching vibration of the methylene group [25]. The C=O asymmetric stretching vibration absorption bands were responsible for the absorption bands at 1651.78 cm^−1^ and 1659.36 cm^−1^ [11,27,28]. The peaks at 1408.34 cm^−1^ and 1408.79 cm^−1^ indicated C–H bending vibrations [29,30]. The characteristic peaks at 1239.77 cm^−1^ and 1231.30 cm^−1^ were ascribed to the stretching of the oxygen bridge (O-O) [31,32]. The broad bands approximately at 1061.54 cm^−1^ and 1069.10 cm^−1^ were ascribed to C-O stretching vibrations of C-O-H or C-O-C groups [28,30]. In addition, the specific absorption bands at 810.53, 810.91, 884.41, and 882.67 cm^−1^ further confirmed the presence of a mannose unit [27,33], which was consistent with the analysis of the HPLC spectrum. In addition, the peaks at 884.41 and 882.67 cm^−1^ indicated the possibility of the presence of a β-glycosidic linkage [27,30]. Despite the similarity in the spectrum, the position and intensity of the absorption peaks suggested that S1 and S2 had different structural features.

### 2.3. NMR Spectroscopy Analysis

**Figure 3:**
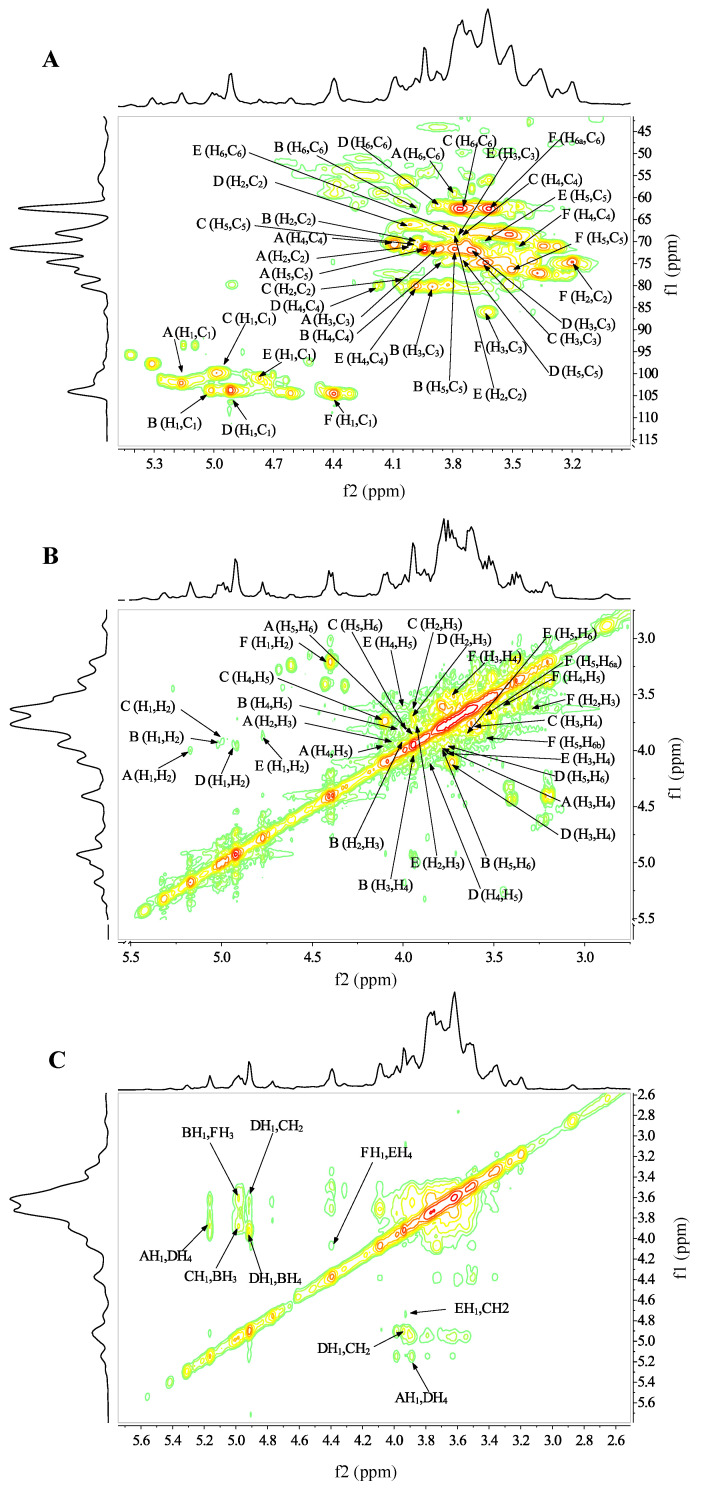
NMR spectrum analysis of S1. (**A**) ^1^H-^13^C HSQC spectrum, (**B**) ^1^H-^1^H COSY spectrum, (**C**) NOESY spectrum.

As shown in the ^1^H NMR spectrum (Figure S2A), S1 exhibited six distinct heterotopic proton signals at *δ* 5.17 (s), 5.01 (s), 4.99 (s), 4.91 (s), 4.77 (s), 4.40 (s, ^3^*J*_H-1, H-2_ = 7.5 Hz) ppm (named A-F), respectively. Based on the coupling constants and the chemical shifts of anomeric hydrogen, residues A-D had *α*-conformations, while residues E-F had *β*-conformations [22]. The 13C NMR spectrum showed the anomeric carbon signals at *δ* 104.52, 103.76, 103.72, 102.11, 100.96, 99.80, and 99.69 ppm (Figure S2B), which further suggested that S1 contained six residues. The absence of C-4 and C-5 signals at *δ* 82-88 ppm showed that the conformation of S1 was pyranotypic. According to the cross-peak signals at *δ* 5.17/102.11 ppm of the HSQC spectrum and *δ* 4.00/3.86 ppm on the COSY spectrum in the heterotopic region (Figure 3A,B), residue A was identified as T-*α*-D-Manp-(1→ [34]. The interaction at *δ* 4.99/99.8 ppm in the heterotopic region of HSQC and the C-2 signal at *δ* 77.51 ppm with downfield shifts suggested that residue C was →2)-*α*-D-Glcp-(1→ [34]. The high-intensity signal of residue D at *δ* 4.91/103.72 ppm on the HSQC spectrum was attributed to →4)-*α*-D-Manp-(1→ [35]. Residue F was inferred as →2)-*β*-D-Glcp-(1→ owing to the downfield shifts of C-2 and the related literature [36]. Using a similar approach, and in conjunction with previous studies, we deduced that residues B and E were →3,4)-*α*-D-Manp-(1→ [37] and →4,6)-*β*-D-Manp-(1→ [38], respectively. The signal at *δ* 67.46 ppm further confirmed the C-6 signal of residue E in the DEPT 135 spectrum (Figure S2B).

The NOESY spectrum showed the signals of connection sites and sequences around the sugar residues of S1 (Figure 3C). The coupling that existed at *δ* 5.17/4.10 ppm (A H_1_/D H_4_) as well as *δ* 4.99/3.88 ppm (C H_1_/B H_3_) demonstrated the existence of A → D and C → B linkages. Moreover, the presence of D → C, E → F, B → F linkages corresponded to the cross-peak signals at *δ* 4.91/3.95 ppm (D H_1_/C H_2_), *δ* 4.40/3.99 ppm (F H_1_/E H_4_), and *δ* 5.01/3.72 ppm (B H_1_/F H_3_), respectively. The above results revealed that the linkage of A → D → C → B → F → E made up the backbone of S1. Additionally, a weak coupling with H1 of residue D (*δ* 4.91 ppm) and H4 of residue B (*δ* 3.93 ppm) suggested that a D → B linkage was present in the side chain.

**Figure 4:**
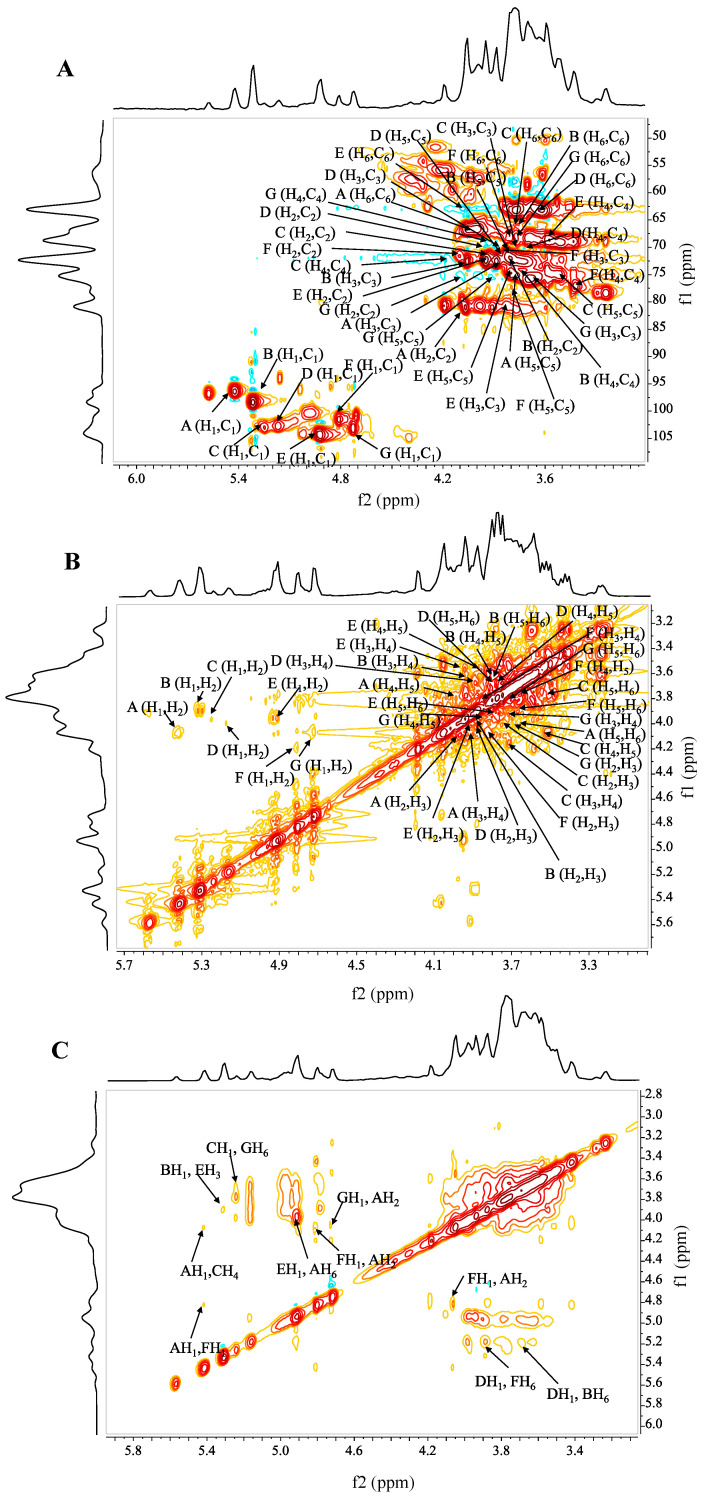
NMR spectrum analysis of S2. (**A**) ^1^H-^13^C HSQC spectrum, (**B**) ^1^H-^1^H COSY spectrum, (**C**) NOESY spectrum.

The ^1^H NMR spectrum of S2 is shown in Figure S3A; seven distinct signals existed at *δ* 5.42 (d, ^3^*J*_H-1, H-2_ = 7.2 Hz), 5.32 (d, ^3^*J*_H-1, H-2_ = 6.8 Hz), 5.25 (s), 5.17 (s), 4.90 (d, ^3^*J*_H-1, H-2_ = 10.5 Hz), 4.81 (d, ^3^*J*_H-1, H-2_ = 9.2 Hz), 4.72 (s) in the heterohead region (named A-G). Combining ^1^H-^13^C HSQC with the ^1^H-^1^H COSY spectrum (Figure 4A,B), the strong cross-peak signals (*δ* 5.42/96.03, 4.06/80.18 ppm), where the C-2 signal had obvious downfield shifts compared with other carbon signals, confirmed that residue A was substituted at C-2. Combined with the substituted C-6 signal (*δ* 66.09 ppm) in DEPT-135 (Figure S3B), it meant that residue A was→2,6)-*α*-D-Manp-(1→ [39]. The strong cross-peak signal (*δ* 5.32/98.32 ppm) of residue B and the signal at C-6 (*δ* 65.6 ppm) were inferred to be →6)-*α*-D-Man*p*-(1→ [34]. Using a similar approach, and combined with literature data [34,40], residues G, E, and C were deduced to be →6)-*β*-D-Gal*p*-(1→, →3)-*β*-D-Man*p*-(1→, →4)-*α*-D-Gal*p*-(1→, respectively. The cross-peak signals (*δ* 5.17/102.41 ppm, *δ* 3.99/69.66 ppm, *δ* 3.89/70.07 ppm, δ 3.66/70.86 ppm) indicated that residue D was *α*-D-Man*p*-(1→ [41]. For residue F, the anomeric signals (*δ* 4.81/101.15 ppm) indicated the existence of a *β*-configuration. The downfield shift of C-4 revealed that the substitution occurred in O-4. Combined with the substituted C-6 (*δ* 67.60 ppm) signal of mannose in DEPT-135, residue F was presumed to be →4,6)-*β*-D-Man*p*-(1→ [38].

The NOESY spectrum of S2 is shown in Figure 4C. The cross-peak signals at *δ* 5.17/3.88 ppm (D H_1_/F H_6_), *δ* 4.81/4.06 ppm (F H_1_/A H_2_), *δ* 5.42/4.12 ppm (A H_1_/C H_4_), *δ* 5.25/3.92 ppm (C H_1_/E H_3_), and *δ* 4.90/3.92 ppm (E H_1_/A H_6_) were identified as the backbone of S2, which consisted of D → F → A → C → E → A linkage. Similarly, the signals at *δ* 5.25/3.71 ppm (C H_1_/G H_6_), *δ* 4.72/4.06 ppm (G H_1_/A H_2_), *δ* 5.17/3.69 ppm (D H_1_/B H_6_), and *δ* 5.32/3.92 ppm (B H_1_/E H_3_) suggested the existence of C → G → A, D → B → E linkages and formed the side chain.

**Figure 5:**
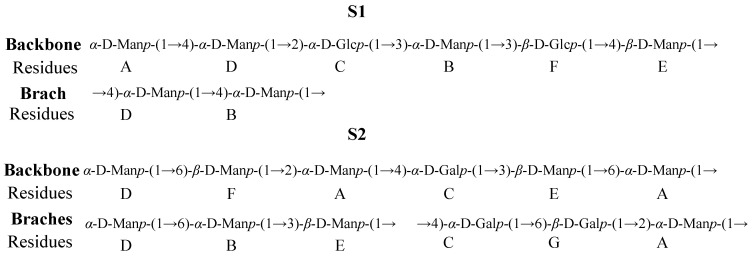
Predicted structures of EPSs.

The structures of S1 and S2 were estimated using the one-dimensional and two-dimensional NMR. The chemical shifts based on the relevant literature are summarized in Table 1 and Table 2. Based on the chemical shifts obtained in NMR analysis, the putative structures of S1 and S2 were proposed (Figure 5), indicating that they were distinct from the structures reported.

### 2.4. SEM Analysis

**Figure 6:**
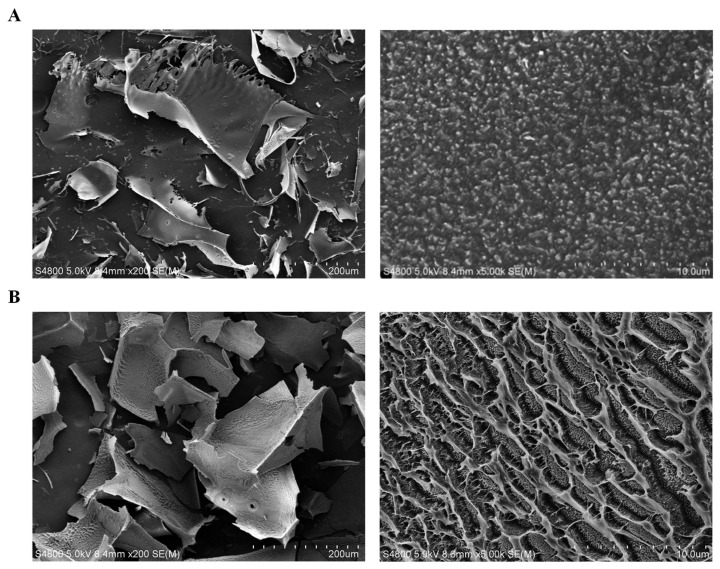
Scanning electron micrograph analysis. (**A**) S1, ×200 (left); ×5000 (right), (**B**) S2, ×200 (left); ×5000 (right).

S1 showed a curly lamellar structure and many pores at ×200 magnification, and the surface was smooth. At ×5000 magnification, the texture was uniform and compact, and the surface was flat (Figure 6A). S2 mainly had a thick sheet structure with a rough surface at ×200 magnification. At ×5000 magnification, the surface of S2 showed a network structure (Figure 6B), which was due to crosslinking and entanglement between polysaccharide molecules.

### 2.5. Thermal Analysis

**Figure 7:**
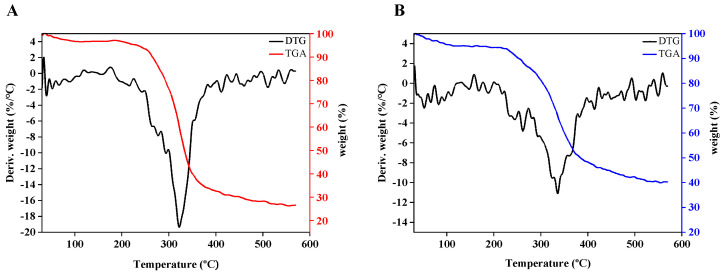
Thermal analysis of EPS. S1 (**A**), S2 (**B**), TGA curves (red line and blue line), DTG curves (black line).

The thermal stability of exopolysaccharides is closely related to their rheological properties. According to the TGA curve analysis, the mass loss trends of the two polysaccharide samples were similar (Figure 7A,B). The mass of S1 decreased more significantly than S2. S1 and S2 exhibited an initial loss of mass as the temperature rose from 30 °C to 110 °C, which was explained by the evaporation of adsorbed water in the biopolymer [42]. DTG curves showed that the mass loss of S1 or S2 occurred mainly at 225−400 °C, which was attributed to the decomposition of the polysaccharide structure, including chemical reactions and changes in functional groups [42]. As the temperature rose from 400 to 570 °C, the masses of S1 and S2 had a slight change. In general, S2 showed great thermal stability, as evaluated by the loss of mass compared with S1, which indicates its potential to be used in the food industry [32,42].

### 2.6. Antioxidant Activity In Vitro

**Figure 8:**
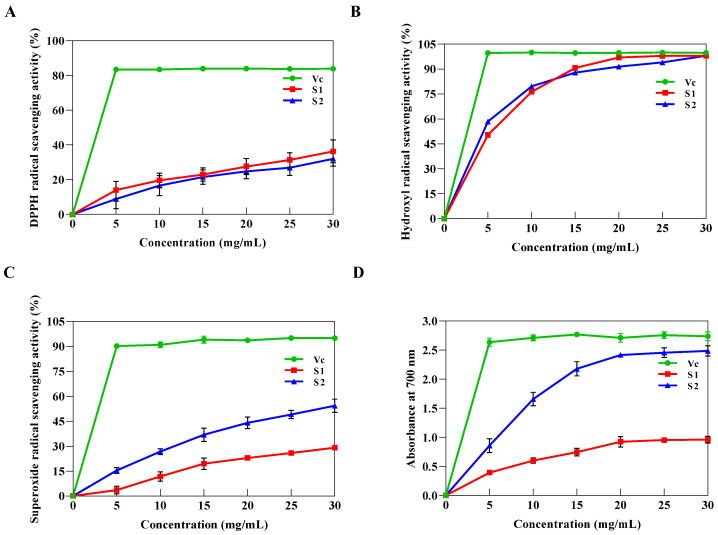
Antioxidant activities of S1 and S2. (**A**) DPPH scavenging activity, (**B**) hydrogen peroxide scavenging activity, (**C**) superoxide radical scavenging activity, (**D**) reducing power.

Free radical scavenging capacity is a key component in the assessment of antioxidant activity. In the experimental concentration range, S1 and S2 exhibited certain DPPH radical scavenging activity and showed a significant dose-dependent increasing pattern, despite being lower than the scavenging activity of Vc (Figure 8A). At the concentration of 5.0 mg/mL, the scavenging activity of S1 and S2 was 14.00 ± 4.14% and 8.89 ± 4.55%, respectively. With the increasing polysaccharide concentration, their scavenging activity was enhanced, reaching 36.26 ± 5.40% and 32.02 ± 3.40% at 30 mg/mL, respectively.

S1 and S2 demonstrated powerful hydroxyl radical scavenging activity that was higher than the majority of reported exopolysaccharides, reaching 98.07 ± 0.15% and 98.07 ± 0.01% at 30 mg/mL, respectively (Figure 8B), which was comparable to Vc [2,30]. Nevertheless, Vc considerably outperformed S1 and S2 in terms of superoxide anion radical scavenging capacity at different concentrations (Figure 8C). S2 displayed good scavenging activity and reached the maximum of 56.58 ± 2.43% at 30 mg/mL compared with S1.

Ferric iron reducing power is positively correlated with absorbance at 700 nm (OD_700_). As shown in Figure 8D, the reducing power was dose-dependent at concentrations ranging from 5 to 20 mg/mL. When the concentration was 30 mg/mL, the absorbance of S1 and S2 reached maximum values of 2.48 ± 0.07 and 0.96 ± 0.05, respectively. Compared to the reducing power of Vc (OD_700_ = 2.75 ± 0.04), S1 and S2 accounted for 90.2% and 34.9% of the relative activity of Vc.

The antioxidant capacity is correlated with the number of electron donors, the structure, and the composition of the polysaccharides [43]. Strong antioxidant properties are often found in polysaccharides that have a larger amount of mannose and glucose and high degrees of branching [22]. Moreover, the lower relative MW of exopolysaccharides generally indicates a stronger antioxidant capacity [32]. It was found that S1 had a weaker antioxidant capacity with a higher MW and a lower degree of branching than S2, which was consistent with the above-reported findings. However, the exact mechanism needs to be further investigated. These results suggested that the two EPSs of *D. Hansenii*, especially S2, have potential to be used as antioxidants in functional foods.

### 2.7. Antitumor Activity In Vitro

**Figure 9:**
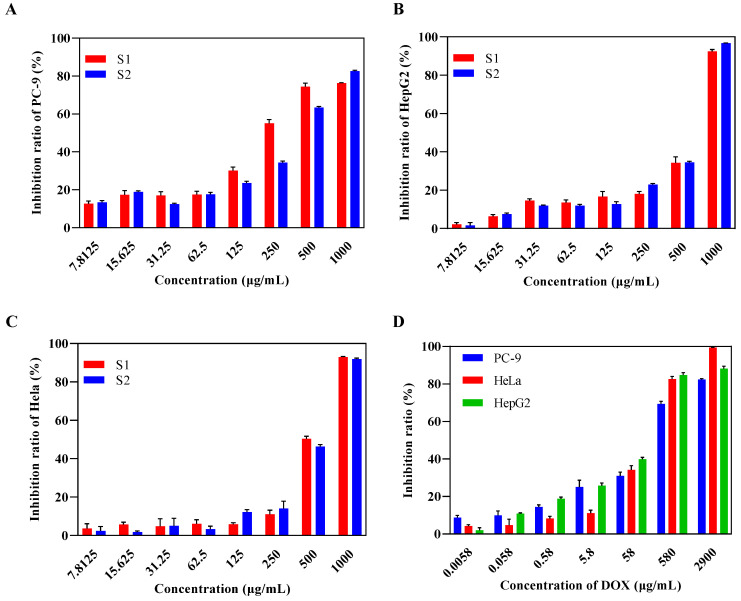
Effects of S1 and S2 on the inhibition ratios of cancer cells PC-9 (**A**), Hela (**B**), and HepG2 (**C**) in vitro, with DOX as a positive control (**D**).

The antitumor activity in vitro against PC-9, Hela, and HepG2 cancer cells was assessed using the CCK8 method (Figure 9). Against the three cell lines, the two new EPSs demonstrated dose-dependent anti-proliferation activity. At the concentration of 1000 μg/mL, S1 and S2 displayed excellent anti-proliferation capacity against all three cell lines, with inhibition rates of more than 80%, especially on Hela and HepG2 cancer cells. The growth inhibition rates of S1 against PC-9, Hela, and HepG2 cancer cells were 74.36 ± 1.50%, 50.26 ± 1.21%, and 34.24 ± 2.58% at the concentration of 500 μg/mL, whereas S2 reached 63.35 ± 0.51%, 46.31 ± 0.90%, and 34.56 ± 0.48%, respectively. This indicated that PC-9 cancer cells appear to be more sensitive to the two novel EPSs compared with Hela and HepG2 cancer cells. In addition, S1 showed better anti-proliferation activity on PC-9 cells than S2 at a low concentration.

Half-maximal inhibitory concentration (IC_50_) values were calculated by GraphPad Prism software based on the non-linear fitting method. As shown in Figure 9D, doxorubicin (DOX) showed great anti-proliferation activity against PC-9, Hela, and HepG2 cancer cells, with IC_50_ values of 118.9, 105.7, and 52.4 μg/mL, while the IC_50_ values of S1 were 222.4, 490.5, and 546.3 μg/mL for PC-9, Hela, and HepG2, and those of S2 were 322.0, 500.2, and 519.3 μg/mL, respectively. Consequently, the results confirmed that PC-9 was more sensitive to S1 compared with S2. In addition, they suggested that S2 displayed a better inhibitory effect on the growth of HepG2 cancer cells due to the lower IC_50_ value compared to S1, but there was no great disparity in the antitumor effects against Hela.

Numerous studies have reported the main antitumor mechanisms of exopolysaccharides, including the following aspects: breaking the cell cycle, inhibiting angiogenesis, inducing the apoptosis of cancer cells, and preserving the immunomodulation system [44,45]. Although there is no relevant literature on the antitumor mechanisms of exopolysaccharides of *D. hansenii*, it was found that the triple helix conformation, the quantity of glucose and mannose, and the degree of branching of polysaccharides were all significant factors in boosting their anticancer activity [45,46]. Polysaccharides with the branches of (1 → 3)-linked and (1 → 6)-linked glycosyl residues tend to exhibit great antitumor activity by promoting contact and interaction with specific receptors [47]. As a result, the excellent antitumor activity of S1 and S2 may be explained by the larger content of mannose or glucose, the triple helix structure, and the moderate (1 → 3)-linked or (1 → 6)-linked glycosidic linkages. Specific mechanisms need to be confirmed by subsequent research. In addition, numerous natural polysaccharides have been found to have both antioxidant and cancer-fighting activity, leading to speculation that there may be a correlation. Although S1 and S2 exhibited strong hydroxyl radical scavenging activity and strong anti-proliferative ability against all three tumor cells, it was noteworthy that the antioxidant capacity of S2 was stronger than that of S1, which was not significantly different from S1 in terms of its ability to inhibit the proliferation of tumor cells. Some studies have suggested that the higher the antioxidant activity, the better the antitumor capacity, yet others have shown that antioxidants are not tumor-inhibiting but rather have a pro-tumor effect. Therefore, a large amount of research is still needed to identify a correlation between them.

## 3. Materials and Methods

### 3.1. Materials and Chemicals

Sausage was provided by Yunnan Dongheng Group Food Co., Ltd. (Jinzhou, China) The DEAE sepharose fast-flow column and Sephacryl S-100 column were obtained from Shanghai Jixing Biotechnology Co., Ltd., Shanghai, China, while 1-phenyl-3-methyl-5-pyrazolone (PMP), 1,1-diphenyl-2-picrylhydrazyl (DPPH), and monosaccharide standards (ribose, xylose, fucose, glucuronic acid, galacturonic acid, mannose, glucose, arabinose, galactose, rhamnose) were from Aladdin Chemistry Company (Shanghai, China). L-ascorbic acid (Vc), trifluoroacetic acid (TFA), and other chemical reagents were obtained from Sinopharm Chemical Reagent Co., Ltd. (Shanghai, China). The three cell lines (PC-9, Hela, HepG2) were provided by Aike Biology Co., Ltd., Qingdao, China.

### 3.2. Screening and Identification of the Strain

A yeast strain producing exopolysaccharides was isolated from sausage using a continuous dilution and plate method. The genome DNA was extracted and then the ITS sequence was amplified with primers ITS4 and ITS1. The obtained nucleotide sequences were searched in the GenBank database. Sequence alignment and analysis, as well as the construction of a phylogenetic tree based on the neighbor-joining (NJ) method, were performed by MEGA software.

### 3.3. Microbial Fermentation and Medium

*D. Hansenii* DH-1 was cultured in the fermentation medium at 30 °C with 180 rpm/min for 96 h, which consisted of the following components (*w*/*v*): 5% glucose, 1% yeast extract, 2% peptone, 5% K_2_HPO_4_, 0.6% MgSO_4_·7H_2_O, 1.0% NaCl, 0.6% (NH_4_)_2_SO_4_, pH 7.5.

### 3.4. Extraction and Purification of EPS

The fermentation broth of *D. Hansenii* DH-1 was centrifugated, evaporated to 1/10 of its original volume, and deproteinated based on the Sevag method using chloroform:n-butyl alcohol (4:1, *v*/*v*). Before collection and freeze-drying, the crude exopolysaccharides were mixed with three times the volume of anhydrous ethanol and stored at 4 °C for 10 h. The samples were sequentially purified using the DEAE sepharose column (1.6 cm × 10 cm) with a step elution gradient from 0 to 1.0 mol/L NaCl solution, and they were further separated through a Sephacryl S-100 column (16 mm × 60 cm). The content of carbohydrates was evaluated by the method of phenol–sulfuric acid colorimetry [21].

### 3.5. Monosaccharide Composition

PMP derivatization was used to analyze the monosaccharide composition [8]. Briefly, 5.0 mg of EPS dissolved in TFA solution (4 mol/L) was hydrolyzed at 105 °C for 6 h, and 100 μL NaOH (0.3 mol/L), as well as 120 μL PMP–methanol solution (0.5 mol/L), was added for the derivatization reaction at 70 °C for 1 h. EPS was extracted with chloroform after being neutralized with HCl (0.3 mol/L). Analysis was performed on a high-performance liquid chromatography system (HPLC, Shimadzu, Japan) using a Shiseido C18 column (4.6 mm × 250 mm, 5 μm).

### 3.6. Molecular Weight Determination

To evaluate the molecular weight (MW) and homogeneity, high-performance gel permeation chromatography (HPGPC, Agilent, Palo Alto, CA, USA) was employed in combination with a TSK G-5000_PWXL_ column (7.8 × 300 mm, 8 μm). MW was estimated with reference to the curves on dextran standards.

### 3.7. Fourier-Transform Infrared Spectroscopy (FT-IR)

Functional groups and glucosidic linkages in compounds were evaluated using an FT-IR spectrometer (Thermo Fisher, Waltham, MA, USA). The EPS was pressed into a 0.1 mm wafer with potassium bromide and examined at 400–4000 cm^−1^.

### 3.8. Congo Red Test

First, 5.0 mg/mL samples were mixed with Congo red solution (80 μmol/L) in equal volumes, and then NaOH was added to reach 0, 0.1, 0.2, 0.3, 0.4, 0.5 mol/L, respectively. After 10 min at ambient temperature, the maximum absorption wavelength (*λ*_max_) was monitored in the 400–600 nm region.

### 3.9. Nuclear Magnetic Resonance (NMR)

First, 50 mg of EPS was deuterium-exchanged by lyophilization three times with D_2_O. Two-dimensional (^1^H-^13^C HSQC, ^1^H-^1^H COSY, and ^1^H-^1^H NOESY) and one-dimensional (^1^H, ^13^C) NMR spectra were recorded on an AVANCE-III 600 MHz NMR spectrometer (Bruker, Switzerland) at 25 °C. The water peaks (*δ*_H_ 4.64 ppm) and chemical shifts of deuterated acetone (*δ*_H_ 2.225 ppm, *δ*_C_ 31.07 ppm) were used for calibration.

### 3.10. Thermal Properties

Under the protection of argon gas, 5.0 mg of EPS was heated at a rate of 20 °C/min from 30 °C to 570 °C. Thermogram analysis (TGA) and differential thermal gravity (DTG) were performed on an SDT-650 simultaneous thermal analyzer (TA, Milford, MI, USA).

### 3.11. Scanning Electron Microscopy (SEM)

The morphology and structure of EPS were micrographed at ×200 and ×5000 magnification and recorded on a Zeiss EVO-18 electron microscope (Oberkochen, Baden-Württemberg, Germany).

### 3.12. Antioxidant Activity Analysis

Samples were diluted to the following concentrations: 0, 5, 10, 15, 20, 25, 30 mg/mL. Vc served as the positive control. The whole experiment was detected using the microplate reader.

#### 3.12.1. DPPH Radical Scavenging Activity

The reaction solution, which contained 1.0 mL of EPS and 4.0 mL of DPPH–ethanol solution (0.004%, *w*/*v*), was placed for 30 min in the dark. The activity was detected at 517 nm and calculated as follows:

(1) $$\mathrm{DPPH}\;\mathrm{scavenging}\;\mathrm{activity}\;\left(\%\right)=\left(1-{\;A}_{1}/A_{0}\right)\times 100$$

where A_0_ represents the absorbance of the control (replaced by absolute ethanol). A_1_ represents the absorbance of the EPS.

#### 3.12.2. Hydroxyl Radical Scavenging Activity

EPS (0.25 mL) was mixed with 1,10-phenanthroline (0.15 mL, 5 mM), sodium phosphate buffer (0.4 mL, 0.75 M, pH 7.4), and FeSO_4_·7H_2_O (0.25 mL, 7.5 mM). After adding 0.1 mL H_2_O_2_ (1%, *v*/*v*), the mixture was incubated at 37 °C for 1 h. The activity was monitored at 510 nm and calculated as follows:

(2) $$\mathrm{Hydroxyl}\;\mathrm{scavenging}\;\mathrm{activity}\;\left(\%\right)=\left(1-\left(A_{2}-A_{1}\right)/A_{0}\right)\times 100\;$$

where A_0_ is the mixed solution of EPS and the reaction solution; A_1_ is the mixed solution without adding EPS (instead of water); A_2_ is the mixed solution without adding H_2_O_2_ (replaced by water).

#### 3.12.3. Superoxide Scavenging Assay

Briefly, 0.2 mL of EPS was incubated with 3 mL of 0.05 M Tris–HCl buffer (pH 8.2) at room temperature for 20 min, before 500 μL of pyrogallol (30 mM) was added. The mixture was accurately reacted for 4 min after stopping the reaction with HCl (0.5 M). The activity was analyzed at 320 nm and calculated as follows:

(3) $$\mathrm{Superoxide}\;\mathrm{scavenging}\;\mathrm{activity}\;\left(\%\right)=\left(1-\left(A_{2}-A_{1}\right)/A_{0}\right)\times 100$$

where A_0_ represents the mixed solution of EPS and the reaction solution; A_1_ represents the mixed solution without adding EPS (instead of water); A_2_ is the mixed solution without adding H_2_O_2_ (replaced by water).

#### 3.12.4. Reducing Power Assay

The solution contained 0.5 mL of phosphate buffer (0.2 mol/L, pH 6.6), EPS, and 1% (*w*/*v*) potassium ferricyanide solution, which were reacted for 20 min at the temperature of 50 °C. After reacting with 0.5 mL of 10% (*w*/*v*) TCA, the solution was centrifuged and mixed with 0.1% (*w*/*v*) ferric chloride solution in a ratio of 5:1. The absorbance was then measured at a wavelength of 700 nm.

### 3.13. Tumor Cell Viability Assay In Vitro

The anti-proliferation activity on PC-9, Hela, and HepG2 in vitro was evaluated using the CCK-8 assay [48], and doxorubicin (Dox) served as a positive control. After the tumor cells (5 × 10^4^ cells/well) were cultured in 96-well plates, each well received 100 μL of EPS at various concentrations (7.8125, 15.625, 31.25, 62.5, 125, 250, 500, 1000 μg/mL) and was incubated for 48 h at 37 °C. After removing the culture medium, 100 μL CCK-8 solution was added and the cells were incubated for 4 h. Each well was scanned at 450 nm by enzyme labeling after incubation. Half-maximal inhibitory concentration (IC_50_) values were obtained by fitting with GraphPad Prism, and the inhibition rate was assessed using the following equation:

(4) $$\mathrm{Inhibition}\;\mathrm{rate}\;\left(\%\right)=\left(1-\frac{A_{x}}{A_{b}}\right)\times 100$$

where A*_b_* is the absorbance of the blank control and A*_x_* is the experimental sample.

### 3.14. Statistical Analysis

All results for three replicates were presented as the mean ± standard deviation (SD). One-way analysis of variance (ANOVA) was performed, and *p* < 0.05 was regarded as statistically significant.

## 4. Conclusions

In this study, two novel EPSs, named S1 and S2, were purified from the fermentation broth of the *D. Hansenii* DH-1 strain through column chromatography. The analysis of the monosaccharide composition showed that S1 was mainly composed of mannose and glucose in a molar ratio of 12.19:1.00. S2 mainly consisted of mannose and galactose in a molar ratio of 4.00:1.00. Mws of S1 and S2 were 34.594 kDa, 24.657 kDa, respectively. Based on FT-IR and NMR data, S1 contained the backbone of α-D-Manp-(1→4)-α-D-Manp-(1→2)-α-D-Glcp-(1→3)-α-D-Manp-(1→3)-β-D-Glcp-(1→4)-β-D-Manp-(1→, and the branch of the →4)-α-D-Manp-(1→4)-α-D-Manp-(1→. S2 mainly contained a backbone fragment of α-D-Manp-(1→6)-β-D-Manp-(1→2)-α-D-Manp-(1→4)-α-D-Galp-(1→3)-β-D-Manp-(1→6)-α-D-Manp-(1→, and the fragments of α-D-Manp-(1→6)-α-D-Manp-(1→3)-β-D-Manp-(1→and→4)-α-D-Galp-(1→6)-β-D-Galp-(1→2)-α-D-Manp-(1→ were present in the side chain. In addition, both S1 and S2 had a triple helix structure, as well as great thermal stability. SEM analysis suggested a distinctive structural difference between S1 and S2. S1 displayed stronger hydroxyl radical scavenging activity and ferric iron reducing power in a dose-dependent manner, while S2 only exhibited strong scavenging activity against hydroxyl radicals. In addition, they significantly inhibited the proliferation of PC-9, Hela, and HepG2 cancer cells, and PC-9 was more sensitive to S1. In summary, S1 and S2 have great potential to be used as antioxidants in the food industry, and they are promising candidates for cancer treatment.

## Figures and Tables

**Table 1:** ^1^H and ^13^C NMR spectrum assignments for S1 (ppm).

Residue	Sugar Linkage		1	2	3	4	5	6
(A)	*α*-D-Man*p*-(1→	H	5.17	4.00	3.86	4.12	3.93	3.83
		C	102.11	70.11	71.55	70.44	70.53	60.71
(B)	→3,4)-*α*-D-Man*p*-(1→	H	5.01	3.95	3.88	3.93	3.78	3.98
		C	103.76	70.08	80.21	76.49	71.60	62.54
(C)	→2)-*α*-D-Glc*p*-(1→	H	4.99	3.95	3.61	3.71	3.97	3.77
		C	99.80	77.51	72.21	68.43	74.12	62.47
(D)	→4)-*α*-D-Man*p*-(1→	H	4.91	3.95	3.69	4.10	3.78	3.71
		C	103.72	67.31	74.72	80.09	74.78	61.69
(E)	→4,6)-*β*-D-Man*p*-(1→	H	4.77	3.86	3.74	3.99	3.66	3.85
		C	100.96	70.34	69.60	80.01	72.34	67.46
(F)	→2)-*β*-D-Glc*p*-(1→	H	4.40	3.21	3.72	3.52	3.57	3.71,3.96
		C	104.52	74.70	82.63	71.44	76.37	62.50

**Table 2:** ^1^H and ^13^C NMR spectrum assignments for S2 (ppm).

Residue	Sugar Linkage		1	2	3	4	5	6
(A)	→2,6)-*α*-D-Man*p*-(1→	H	5.42	4.06	3.99	3.80	3.99	3.92
		C	96.03	80.18	72.17	74.84	74.34	67.10
(B)	→6)-*α*-D-Man*p*-(1→	H	5.32	3.87	3.96	3.60	3.80	3.69
		C	98.32	71.76	73.41	75.01	71.33	65.6
(C)	→4)-*α*-D-Gal*p*-(1→	H	5.25	3.96	3.70	4.12	3.51	3.74
		C	102.68	71.99	70.28	72.25	73.71	65.62
(D)	T-*α*-D-Man*p*-(1→	H	5.17	3.99	3.89	3.66	3.76	3.62
		C	102.41	69.66	70.07	70.86	70.42	62.89
(E)	→3)-*β*-D-Man*p*-(1→	H	4.90	3.94	3.92	3.58	3.86	3.67/3.90
		C	103.54	71.86	80.46	68.4	75.2	62.16
(F)	→4,6)-*β*-D-Man*p*-(1→	H	4.81	4.19	3.82	3.75	3.66	3.88
		C	101.15	71.23	70.07	76.76	71.10	67.6
(G)	→6)-*β*-D-Gal*p*-(1→	H	4.72	4.05	3.68	3.92	3.91	3.71
		C	103.5	74.84	75.19	68.40	75.47	67.82

## Data Availability

The data presented in this study are available in the article.

## Supplementary Materials

The following supporting information can be downloaded at: https://www.mdpi.com/article/10.3390/ijms24010335/s1.
