# Supplementary material for: Structural Characterization, Antioxidant and Antitumor Activities of the Two Novel Exopolysaccharides Produced by Debaryomyces hansenii DH-1
Source: Int J Mol Sci. 2022 Dec 25;24(1):335. doi: 10.3390/ijms24010335 (PMC9820826; doi:10.3390/ijms24010335)
Supplement: Supplementary file 1 [file ijms-24-00335-s001.zip › ijms-2108069-supplementary.pdf]

# Supplementary Materials:

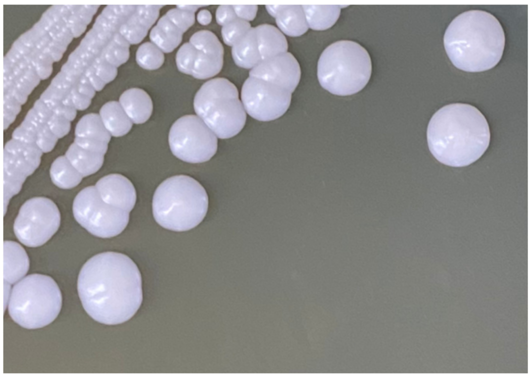

**Figure S1.** Growth morphology of *D. Hansenii* DH-1 on agar plate.

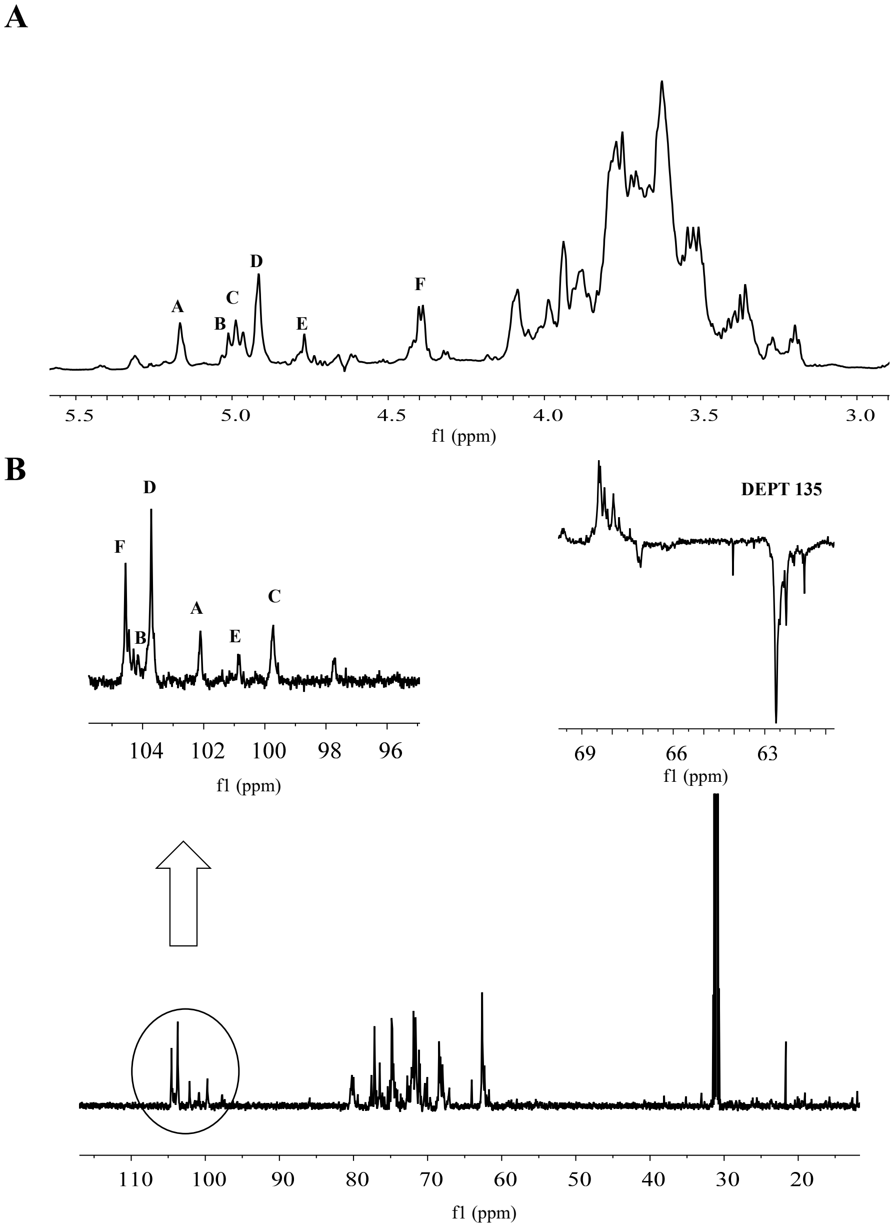


**Figure S2.** NMR spectrum analysis of S1. (A) $^{1}H$ spectrum, (B) $^{13}C$ spectrum.

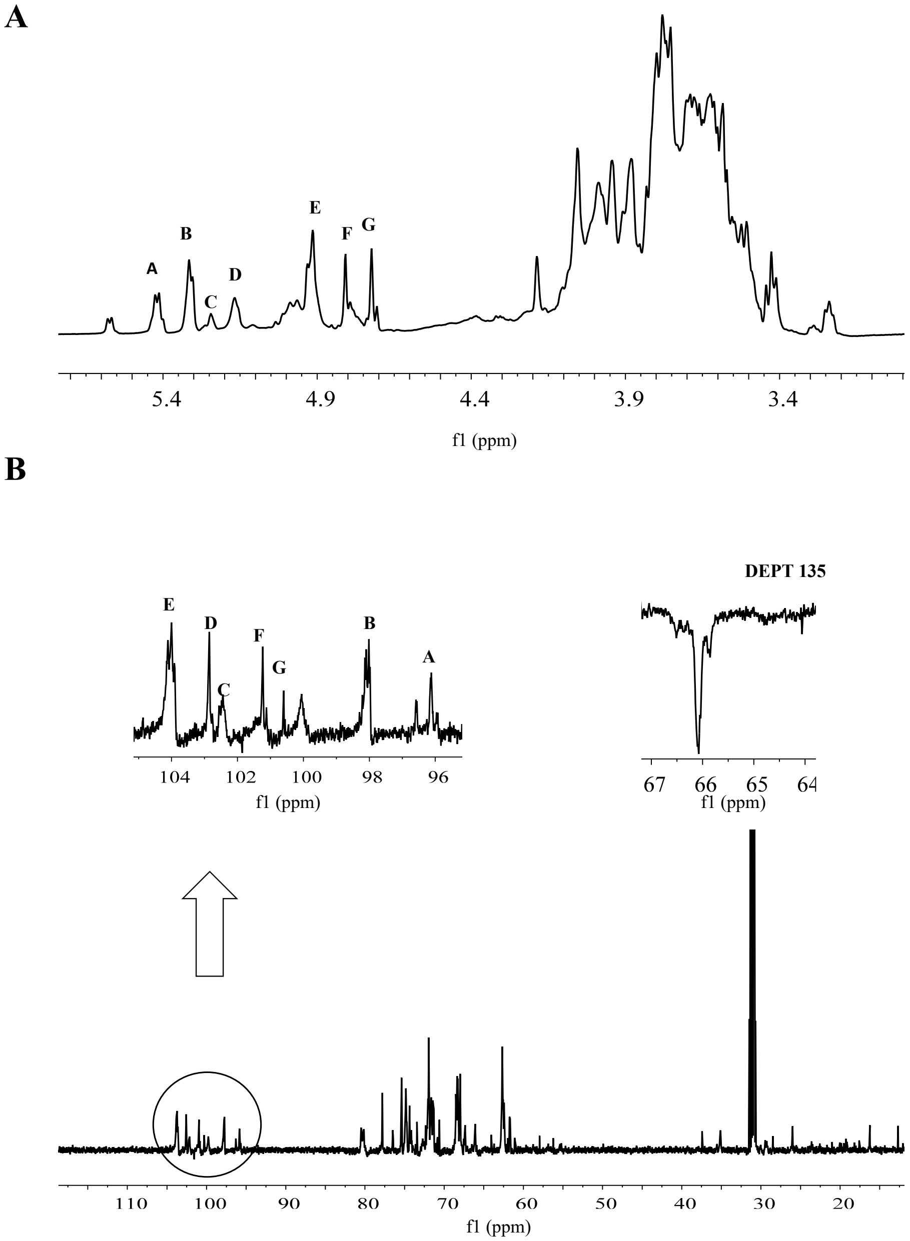


**Figure S3.** NMR spectrum analysis of S2. (A) $^{1}$H spectrum, (B) $^{13}$C spectrum.

**Table S1.** Molecular Weight Averages of EPSs.

| Polysaccharides | Mp (g/mol) | Mn (g/mol) | Mw (g/mol) | Mz (g/mol) | Mz+1 (g/mol) |
|---|---|---|---|---|---|
| S1 | 13869 | 13655 | 34594 | 138813 | 321152 |
| S2 | 8833 | 12988 | 24657 | 91134 | 255994 |
